# Distinct Effects of IL-18 on the Engraftment and Function of Human Effector CD8^+^ T Cells and Regulatory T Cells

**DOI:** 10.1371/journal.pone.0003289

**Published:** 2008-09-26

**Authors:** Richard G. Carroll, Carmine Carpenito, Xiaochuan Shan, Gwenn Danet-Desnoyers, Ronghua Liu, Shuguang Jiang, Steven M. Albelda, Tatiana Golovina, George Coukos, James L. Riley, Zdenka L. Jonak, Carl H. June

**Affiliations:** 1 Abramson Family Cancer Research Institute, University of Pennsylvania School of Medicine, Philadelphia, Pennsylvania, United States of America; 2 Department of Pathology and Laboratory Medicine, University of Pennsylvania School of Medicine, Philadelphia, Pennsylvania, United States of America; 3 Department of Medicine, University of Pennsylvania School of Medicine, Philadelphia, Pennsylvania, United States of America; 4 Department of Obstetrics and Gynecology, University of Pennsylvania School of Medicine, Philadelphia, Pennsylvania, United States of America; 5 GlaxoSmithKline, Biopharm-CEDD, Biology US, King of Prussia, Pennsylvania, United States of America; New York University School of Medicine, United States of America

## Abstract

IL-18 has pleotropic effects on the activation of T cells during antigen presentation. We investigated the effects of human IL-18 on the engraftment and function of human T cell subsets in xenograft mouse models. IL-18 enhanced the engraftment of human CD8^+^ effector T cells and promoted the development of xenogeneic graft versus host disease (GVHD). In marked contrast, IL-18 had reciprocal effects on the engraftment of CD4^+^CD25^+^Foxp3^+^ regulatory T cells (Tregs) in the xenografted mice. Adoptive transfer experiments indicated that IL-18 prevented the suppressive effects of Tregs on the development of xenogeneic GVHD. The IL-18 results were robust as they were observed in two different mouse strains. In addition, the effects of IL-18 were systemic as IL-18 promoted engraftment and persistence of human effector T cells and decreased Tregs in peripheral blood, peritoneal cavity, spleen and liver. In vitro experiments indicated that the expression of the IL-18Rα was induced on both CD4 and CD8 effector T cells and Tregs, and that the duration of expression was less sustained on Tregs. These preclinical data suggest that human IL-18 may have use as an adjuvant for immune reconstitution after cytotoxic therapies, and to augment adoptive immunotherapy, donor leukocyte infusions, and vaccine strategies.

## Introduction

Initially isolated from macrophages as a potent interferon-γ (IFN-γ)-inducing factor [1], [2], Interleukin-18 (IL-18) is a multifunctional cytokine affecting both innate and adaptive immune responses [3], [4]. IL-18 is produced as an inactive precursor (pro-IL-18), which is activated by proteolytic cleavage, primarily by caspase-1 [5], [6]. Pro-IL-18 is produced by multiple cell types, including macrophages [1], dendritic cells [7], vascular endothelial cells [8], and intestinal epithelial cells [9].

Numerous effector functions have been attributed to IL-18. Perhaps the best characterized of these is IL-18's context-dependent induction of Th1 or Th2 CD4 T cell polarization [3]. In the presence of IL-12, IL-18 drives the Th1 polarization of activated CD4^+^ T cells. However, in the absence of IL-12 (or in the presence of IL-2 or IL-4), IL-18 promotes IgE expression and Th2 differentiation. More recently, IL-18 has been demonstrated, in synergy with IL-23, to drive TH17 cell polarization [10], [11]. IL-18 also exerts multiple effects on NK cells, including increased cytoxicity [12], and in synergy with IL-2, promotion of NK cell expansion and IFNγ production [13]. Given its widespread immune-augmenting functions, it is not surprising that IL-18 has been evaluated in numerous animal models of cancer. For example, IL-18 enhances the efficacy of DNA vaccines directed against prostate-specific antigen [14] or Fos-related antigen [15]. Additionally, IL-18 augments the efficacy of DC-based vaccines [16], [17] as well as whole-cell tumor vaccines [18]. However, IL-18 has also been reported to enhance tumor progression (reviewed in [19]). For example, tumor-produced IL-18 induces Fas ligand expression in melanoma cells, possibly resulting in escape from NK cell-mediated immune surveillance [20]. Furthermore, IL-18 has been shown to increase the invasiveness of myeloid leukemia lines [21].

IL-18 has also been implicated in multiple autoimmune-associated pathologies (reviewed in [22]). For example, high levels of IL-18 are found in the synovial fluid of rheumatoid arthritis patients [23], and alleviation of rheumatoid arthritis symptoms is associated with a decrease in IL-18 levels [24]. Paradoxically, given its' proinflammatory properties, IL-18 is well tolerated and safe in humans [25]. In contrast, IL-12 is toxic at doses three orders of magnitude lower [26].

The rationale for the present study was to determine if IL-18 might present a less toxic alternative to IL-12 as an adjunct for cancer adoptive transfer immunotherapy. In the study described herein, we demonstrate that IL-18 administration resulted in increased engraftment of CD8^+^ human T cells. Concurrently, IL-18 administration resulted in a decrease in human CD4^+^CD25^+^FoxP3^+^ regulatory T cells (Tregs). Furthermore, we find that IL-18 augmented xenogeneic GVHD, and overrode the suppressive effect of Tregs *in vivo*. Our findings indicate that by simultaneously affecting both CD8^+^ T cells and Tregs, IL-18 may alter the set point of an immune response, underscoring the potential utility of IL-18 as an adjuvant in cancer therapy.

## Methods

### Mice

All animal experiments were approved by the University of Pennsylvania Institutional Animal Care and Use Committee. NOD/*scid*/β2microglobulin*^null^* (NOD/*scid*/β2*m^null^*) [27] and NOD/*scid*/IL-2rγ*^null^* (NOG) [28] mice were purchased from the Jackson Laboratory. Animals were bred in the Animal Services Unit of the University of Pennsylvania. The mice were housed under specific pathogen-free conditions in microisolator cages and given unrestricted access to autoclaved food and acidified water. Animals of both sexes were used for experiments at 6–9 weeks of age.

### Cells and Animal Injections

Peripheral blood mononuclear cells (PBMCs) were obtained by leukapheresis of healthy volunteer donors by the University of Pennsylvania Human Immunology Core. All specimens were collected under a University Institutional Review Board-approved protocol, and informed consent was obtained from each donor. Cells were injected intraperitoneally into non-irradiated host animals as described in the individual experiments.

Recombinant human IL-18 (SB-485232) and pegylated human IL-18 (GSK-189720) were prepared as previously described at GlaxoSmithKline [29]. Subcutaneous injections of 15 µg/animal were performed daily for the recombinant product and twice weekly for the pegylated product, taking into consideration the increased persistence of the pegylated IL-18.

### Animal Necropsy

Animals were sacrificed by CO_2_ asphyxiation. Peripheral blood was obtained by cardiac puncture, and the spleen, liver, lung, and gut were isolated and portions were fixed in 4% paraformaldehyde. Liver leukocytes were separated by Ficoll density gradient centrifugation and splenocytes were prepared by mechanical disassociation. Cells were retrieved from the peritoneal cavity by PBS lavage.

### Immunohistochemistry

Mouse tissues were fixed in 4% paraformaldehyde and embedded in paraffin. 5–6 µm sections were cut for immunohistochemical staining. Following deparaffinization and high temperature antigen unmasking procedures, sections were incubated with murine monoclonal antibodies to human CD25 (Clone 4C9 [Vector], 1∶100), or Foxp3 (Clone236A/E7 [AbCam], 1∶40). Sections were then incubated with biotinylated secondary antibody (goat anti-mouse IgG [Vector]) and signal was localized using 3,3′-diaminobenzidine tetrahydrochloride (DAB, [Vector]) as the chromogen. Hematoxylin was used for counterstaining. Positively stained cells were quantified with Metamorph software (Universal Imaging Corporation) using the Manual Count option. Quantification was performed in a blinded manner. For each organ, at least 8 fields per animal were tabulated.

### Flow cytometry

After euthanasia, peripheral blood, peritoneal cavity washings, spleen, and liver were obtained from euthanized animals. Single cell suspensions were stained with conjugated anti-human lymphocyte surface markers (CD45, clones H130 or 2D1, CD4, clone SK3, CD8, clone SK1 or 3B5, and CD25, clone M-A251, all from BD Pharmingen) and Foxp3 (Clone 206D, Biolegend). Cell populations were analyzed using either LSRII or FACSCalibur flow cytometers (Becton Dickinson) and Flowjo software (Tree Star). Human lymphocytes were initially identified by gating on the human CD45^+^ population within the live gate, and human lymphocyte subsets were further defined within this population. The absolute number of human cells was determined using TruCount tubes (BD Biosciences) or a Multisizer 3 (Beckman Coulter).

### Adoptive Transfer of Ex Vivo Expanded Tregs

Human CD4^+^CD25^high^ cells were isolated from PBMCs using magnetic beads (Miltenyi Biotech) as described [30]. Gamma-irradiated (100 Gy) K562-based antigen presenting cells modified to express CD64, CD86 and OX40L [31] were used to stimulate purified populations of CD4^+^CD25^high^ cells, which were expanded ex vivo in the presence of 300 U/ml IL-2 (Chiron) and 100 ng/ml Rapamycin (Calbiochem) [30]. After one growth cycle (approximately 14–21 days), the cells were harvested and used for injection as described in the text.

### *In Vitro* Suppression Assay

Following harvest of expanded T cells (Tregs and control CD4 cells), varying numbers of Tregs were plated in 100 µl of X-VIVO 15+10% human AB serum in round bottom 96 well plates (Corning Incorporated, Corning, NY). Frozen autologous PBMCs were thawed, CFSE-labeled, and resuspended in culture medium at 1×10^7^/ml. Anti-CD3 beads (Invitrogen) were added at a ratio of 1 bead per cell. 100 µl of PBMC cell suspension (1×10^6^ cells) was added to individual wells in a 96 well plate. CFSE-labeled PBMCs without CD3 beads (and lacking Tregs) were used as negative controls; CFSE-labeled PBMC stimulated with anti-CD3 beads (no Tregs) were used as positive controls. Where indicated, the suppression assay was performed in the presence of 500 ng/ml recombinant human IL-18. The cultures were harvested four days later and stained with APC-conjugated anti-CD8 (BD Pharmingen). Data were acquired on a FACSCalibur flow cytometer using Cell Quest Pro software and analyzed using FlowJo software. Quantitative analysis of Treg suppression capacity was performed by gating on CD8^+^ CFSE^+^ cells [30].

### Statistical analysis

All results were expressed as means±standard deviation. Unless otherwise indicated, the statistical significance of differences between groups was evaluated by two-sample t tests assuming equal variance or the Mann-Whitney rank sum test. Survival data were analyzed by lifetable methods using log rank analysis performed using SysStat (Systat Software, Inc.).

## Results

**Figure 1:**
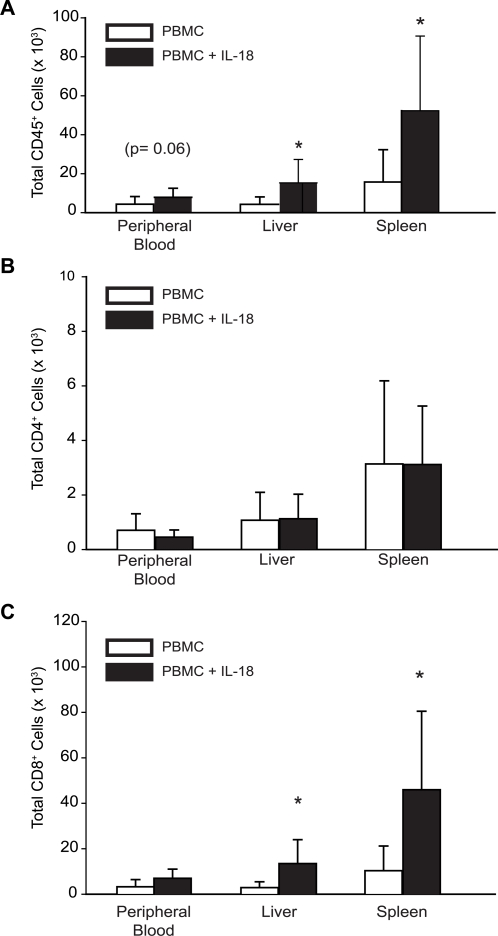
Human IL-18 Promotes Systemic Increases in Human CD8 T Cell Numbers in NOD/*scid/*β2*m* ^null^ mice injected with human PBMCs. NOD/*scid*/β2*m^null^* mice were injected intraperitoneally with 5×10^7^ human PBMCs and 24 hours later, a three week course of daily subcutaneous injections of 15 µg recombinant human IL-18 was initiated. At the end of the injection time course, the animals were sacrificed, peripheral blood was collected, and liver and spleen homogenates were prepared. Human cell populations were detected by flow cytometry. Absolute cell numbers in the peripheral blood were determined using Tru-Count tubes; absolute cell numbers from spleen and liver were determined using a Coulter Multisizer 3. The mean±s.d. is shown for (A) human CD45^+^ cells, (B) human CD4^+^ cells, and (C) human CD8^+^ cells. Open bars represent values from mock-treated animals; filled bars represent values from IL-18 treated animals. These data are a representative single experiment of seven different experiments. Asterisks indicate significant differences between mock- and IL-18-treated animals (n = 6 to 10 mice/group; p<0.05).

In preliminary experiments we evaluated recombinant human IL-18 at doses from 1 to 100 µg/mouse/day for anti-tumor effects in xenografted mice (data not shown), and 15 µg was chosen as a daily dose based on tolerability and efficacy, and because this dose is less than the equivalent maximum tolerated dose that has been established in monkeys and humans [25], [32]. The effects of IL-18 were tested on human T cell subsets using two humanized mouse models [27], [28]. Initially, we determined if IL-18 directly affected the engraftment of human T cells in NOD/*scid*/β2*m^null^* mice. These mice, in addition to lacking mature B and T cells, have reduced levels of NK cells compared to NOD/*scid* mice [27]. When NOD/*scid*/β2*m^null^* mice were injected intraperitoneally with PBMC, human CD45 positive cells were recovered from peripheral blood, liver and spleen. Daily subcutaneous injections of IL-18 caused a substantial increase in the number of human leukocytes recovered from the blood, liver and spleens of the mice (**Fig. 1A**). However, the leukocyte promoting effect of IL-18 was entirely attributable to increased engraftment of CD8^+^ T cells, as the number of CD4^+^ T cells was not increased by IL-18 (**Fig. 1B and 1C**). No NK cells or B cells were observed at this time point (data not shown).

**Figure 2:**
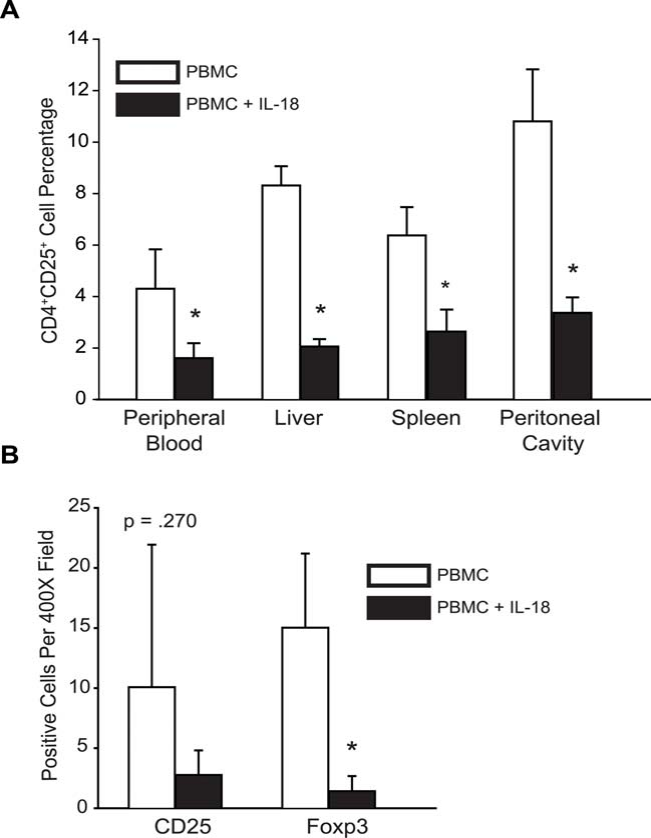
Human IL-18 Mediates a Decrease in Tregs in NOD/*scid/*β2*m^null^* mice Injected with Human PBMCs. 5×10^7^ PBMCs were transferred into NOD/*scid* /β2M*^null^* mice. The day following cell injection, a three week course of subcutaneous huIL-18 injections was initiated. Animals in the experiment depicted in panel A received twice-weekly doses of 15 µg pegylated human IL-18, while the animals in the experiments depicted in panel B received daily doses of 15 µg recombinant human IL-18. (A) At the end of IL-18 treatment, cells isolated from the peripheral blood, peritoneal cavity, spleen and liver were stained with antibodies to human CD45, CD4, and CD25 and analyzed by flow cytometry. The mean percentage of CD^+^CD25^+^ cells from each tissue is depicted. Filled bars represent values obtained from IL-18-treated animals; open bars represent values obtained from mock-treated animals. (B) Paraffin sections from the spleens of mock- or IL-18-treated animals were reacted with antibodies specific for human CD25 or Foxp3 . Antibody-reactive cells per 400X field were enumerated blindly, and the mean±s.d. depicted. Solid bars depict sections from IL-18-treated animals, while open bars represent sections from mock-treated animals. The experiment in panel A was performed 2 times, and the experiment in panel B was performed 6 times. Asterisks indicate significant differences between mock- and IL-18-treated animals (n = 8/group; p<0.05).

As is shown below, we observed that the IL-18 treated mice developed xenogeneic GVHD more rapidly than mice engrafted with PBMC only. We first considered the hypothesis that the IL-18-mediated increase in human CD8^+^ cells could account for this. However, an alternative hypothesis was also considered, that decreased engraftment with regulatory T cells could also account for this, because others have shown that regulatory T cell numbers and function can influence the onset and severity of xenogeneic GVHD [33], [34]. Although IL-18 did not influence the total number of CD4 cells (**Fig. 1B**), there were significant decreases in regulatory T cells in the IL-18 treated mice, as judged by CD25 and FoxP3 expression (**Fig. 2**). The percentage and absolute number of CD4^+^CD25^+^ cells was decreased in all organs and tissues examined, i.e. blood, spleen, liver, and peritoneal cavity (**Fig. 2A and 2B**).

**Figure 3:**
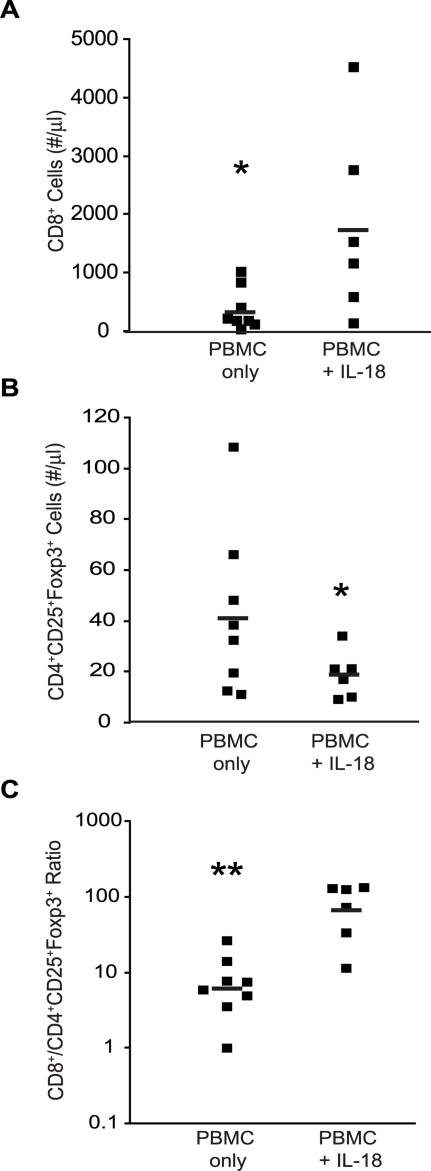
The Human IL-18-Mediated Decrease in Tregs is Not Restricted to NOD/*scid/*β2*m^null^* Mice. NOD/*scid*/IL-2rγ*^null^* (NOG) mice were injected with 5×10^6^ human PBMCs, and 24 hours later, a three week course of daily subcutaneous injections of 15 µg recombinant human IL-18 was initiated. The absolute numbers of human CD8^+^ cells (A) and CD4^+^CD25^+^Foxp3^+^ cells (B) in the peripheral blood were determined using TruCount tubes. Values from individual mice are depicted. (C) The ratio of CD8 cell number to CD4^+^25^+^Foxp3^+^ cell number for each animal in panels A and B is plotted on a log scale. In panels A and B, asterisks (*) indicate significant differences between mock- and IL-18-treated animals (n = 6 to 8/group; p<0.05). In panel C, asterisks (**) indicate p<0.001.

It was possible that the distinct effects of IL-18 on human lymphocyte subsets were due to relative efficiencies of engraftment for effector and regulatory subsets in the NOD/*scid*/β2*m^null^* mice. To test this possibility, PBMCs were transferred into NOD/*scid*/IL-2rγ*^null^* (NOG) mice. This strain is highly permissive to human hematopoietic stem cell engraftment [28]. In NOG mice injected with PMBC, the mice treated with IL-18 had consistently elevated numbers of human CD45^+^ cells in the peripheral blood, and this increase was entirely accounted for by increased numbers of CD8^+^ cells (**Fig. 3A**) as was the case for NOD/*scid*/β2M*^null^* mice. The NOG mice also had reciprocal decreases in CD4^+^CD25^+^Foxp3^+^ cells in the blood (**Fig. 3B**) and all tissues examined (data not shown). The IL-18 mediated decrease in CD4^+^CD25^+^FoxP3^+^ cells was not due to decreased recovery of the cells, as tissue sections examined by immunohistochemistry also documented consistent decreases in cells that stained for Foxp3 (data not shown). Furthermore, as shown in **Fig. 3C**, the CD8:Treg ratios in the peripheral blood of individual animals were markedly increased by IL-18 treatment.

**Figure 4:**
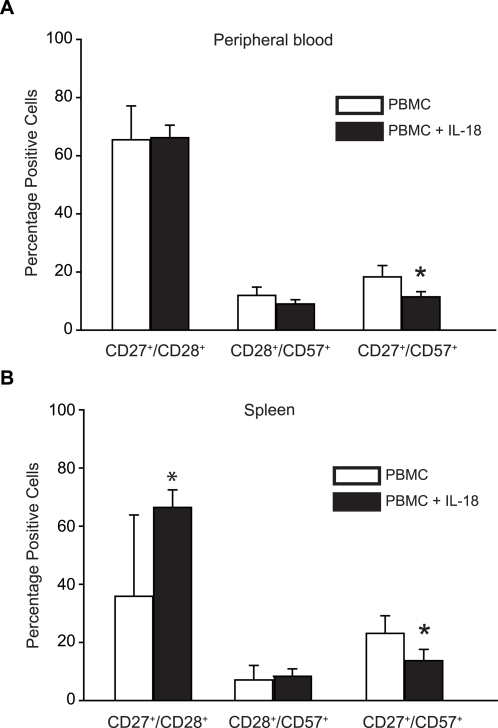
Effects of Human IL-18 on CD8 T Cell Differentiation in NOG mice. NOG mice were injected intraperitoneally with 5×10^6^ human PBMCs, and beginning 1 day later, were injected with 15 µg recombinant human IL-18 daily for three weeks. Spleen tissue and peripheral blood were collected and analyzed for the presence of different CD8^+^ T cell subsets by flow cytometry. Cell populations were initially gated on human CD45 and human CD8. Filled bars represent values mean±s.d. obtained from IL-18-treated animals; open bars represent values obtained from mock-treated animals. The data in this figure represent results from one of two independent experiments. Asterisks indicate significant differences between mock- and IL-18-treated animals (p<0.05).

To assess the effects of IL-18 treatment on human T cell differentiation in NOG mice after IL-18 treatment, peripheral blood and spleen were examined by flow cytometry (**Fig. 4**). There were only minor effects of IL-18 on the fraction of T cells with a central memory (CD27^+^CD28^+^) phenotype, and the modest effects were different in blood and spleen. There was only a slight effect of IL-18 on the appearance of T cells with a senescent or effector memory phenotype (CD28^+^CD57^+^ or CD27^+^CD57^+^).

**Figure 5:**
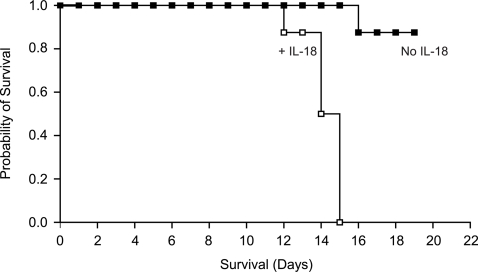
Human IL-18 Accelerates Xenogeneic GVHD in NOG mice. 5×10^6^ PBMC were transferred into NOG mice on day 0, and IL-18 or PBS injected daily starting on day 1. On the x-axis are days after transfer of cells. On the y-axis is the proportion of recipients surviving (n = 8/group; P<.02).

As was mentioned above, we observed that both the NOD/*scid*/β2M*^null^* and NOG mouse strains had earlier onset and more severe xenogeneic GVHD after PBMC engraftment and human IL-18 treatment (**Fig. 5**). Clinical appearance and examination of weight curves revealed that IL-18 treated mice had more significant manifestations of GVHD as indicated by lethargy, hunched posture, generalized erythema, and a progressive reduction in mean weights from pretransplant levels (data not shown). Pathologic examination revealed that the IL-18 treated mice had more severe tissue infiltration and inflammation of the lung, liver and gastrointestinal tracts (**Supplemental Fig. S1** **and** **S2**).

**Figure 6:**
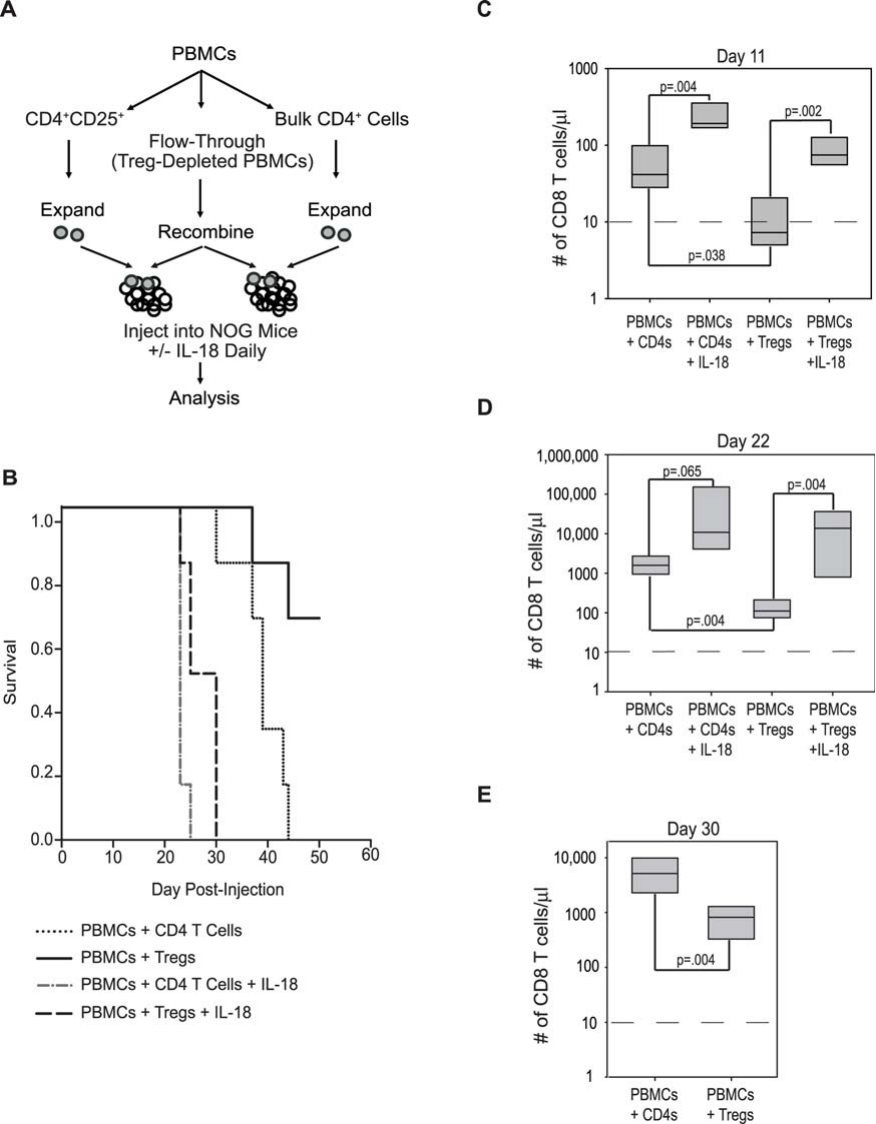
Effect of Human IL-18 on Treg-Induced Delay of Xenogeneic GVHD in NOG Mice. NOG mice were injected with 2×10^7^ Treg-depleted human PBMCs supplemented with either 4 million autologous CD4^+^CD25^−^ T cells (“CD4 T cells”) or 4 million autologous, ex vivo-expanded CD4^+^CD25^+^ cells (“Tregs”). One cohort of each group received daily injections of 15 µg recombinant human IL-18 for three weeks. Human cell engraftment in the peripheral blood was measured 11, 22, and 30 days post-injection. The animals were followed until the onset of xenogeneic GVHD. (A) Experimental overview. (B) Kaplan-Meier Survival Analysis (Log-Rank) of the indicated cohorts of mice. The Holm-Sidak method for multiple comparisons (significance level 0.05) was performed, and showed significant differences between all groups. (C–E) Peripheral blood levels of human CD8^+^ T cells were measured at Day 11 (C), Day 22 (D), and Day 30 (E) post-injection. Note that all IL-18-treated animals are not represented in panel E due to their death from acute GVHD.

To address the mechanisms for these observations, we used an adoptive transfer model of effector and regulatory T cells that we and others have developed [35]–[38]. PBMCs were subjected to immunoaffinity bead separation to obtain CD4^+^25^high^ cells and PBMC depleted of CD4^+^25^high^ cells. The CD4^+^25^high^ cells at baseline were >70% Foxp3^+^, and these cells were expanded using K562 cells expressing CD64, CD86, and OX40L in the presence of IL-2 and rapamycin as described [37]. NOG mice were injected with PBMC plus enriched CD4^+^CD25^−^ effector cells, or with PBMC plus *ex vivo*-expanded CD4^+^25^+^ Tregs. IL-18 treatment *in vivo* accelerated GVHD lethality in mice engrafted with PBMC plus enriched CD4^+^CD25^−^ effector cells, or with PBMC plus *ex vivo* expanded CD4^+^25^+^ cells (**Fig. 6A and B**). In contrast, most mice engrafted with PBMC and *ex vivo* expanded Tregs had long term survival. The mice were monitored for engraftment of CD8^+^ T cells by periodic measurements of absolute CD8^+^ T cell numbers in peripheral blood using the Trucount assay (**Fig. 6C–E**). Mice engrafted with PBMC and CD4^+^ effector cells had a progressive increase in the number of circulating CD8^+^ T cells. Previous studies have shown that human Tregs prevent the expansion of the presumably xenoreactive CD8^+^ T cells in mice [34]. We found that *ex vivo* expanded Tregs also prevented the expansion of CD8^+^ T cells in the mice. However, IL-18 treatment resulted in a striking increase in the numbers of CD8^+^ T cells in mice engrafted with either PBMCs and CD4^+^ effectors or with PBMCs and Tregs. In fact, all IL-18-treated animals were sacrificed early due to acute GVHD symptoms (**Fig. 6E** and data not shown). Thus, in the presence of IL-18, Tregs failed to control CD8+ T cell proliferation. However, the Kaplan-Meier curves show a significant difference between each of the 4 groups, indicating that Tregs have an effect even in the face of IL18 treatment.

**Figure 7:**
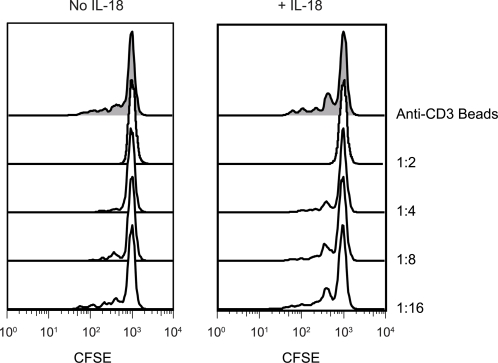
Human IL-18 Does Not Prevent Treg Suppressive Function in Vitro. Purified Tregs were expanded ex vivo for 18 days, and then tested in an in vitro suppression assay. Autologous PBMCs were labeled with CFSE and stimulated using anti-CD3 Ab coated beads and mixed with either no Tregs (top panel) or expanded Tregs at the indicated ratio (Treg:PBMC) in the presence or absence of recombinant human IL-18 (500 ng/ml). Histograms show the expansion of CD8^+^ cells on day 4 of culture.

**Figure 8:**
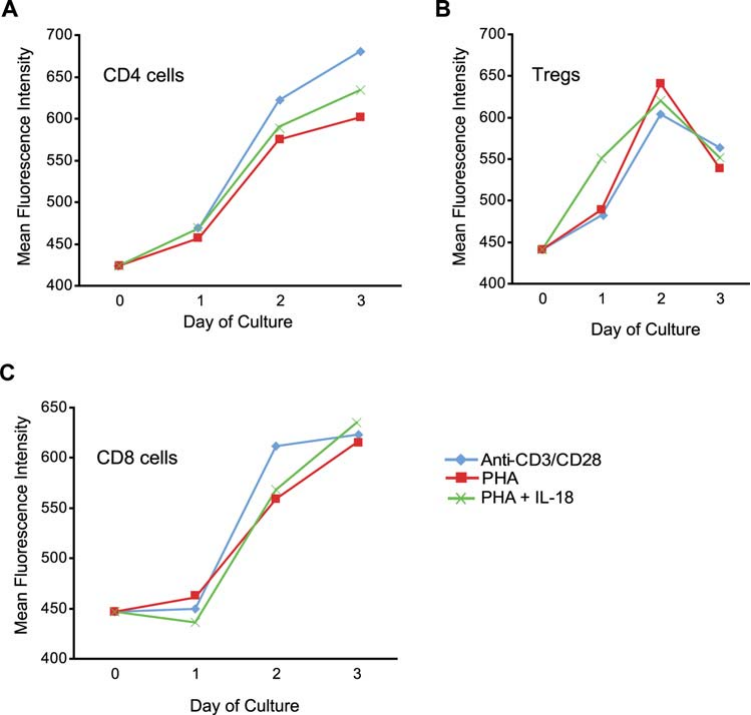
Rapid Down Regulation of IL18-Rα on Tregs Following Cell Activation. Purified populations of Tregs, as well as bulk CD4 and CD8 cells, were activated with either αCD3/αCD28 beads, PHA (5 µg/ml), or PHA+recombinant human IL-18 (500 ng/ml). IL-18Rα expression was measured by flow cytometry. IL-18Rα MFI on effector CD4 cells (A), Tregs (B) and effector CD8 cells (C) is plotted before stimulation and 1, 2, and 3 days following stimulation.

Because IL-18 was able to override some of the effects of the Tregs as indicated by the findings of increased CD8^+^ T cells in mice given adoptively transferred Tregs, we next evaluated the effects of IL-18 on Treg function *in vitro*. We found that the addition of IL-18 to culture medium did not affect the population doubling rate of Tregs *in vitro* after anti-CD3/CD28 stimulation, in the presence or absence of IL-2 (data not shown). Furthermore, IL-18 did not affect the suppressive function of *ex vivo* expanded Tregs (**Fig. 7**) or the expression of Foxp3 (data not shown). Finally, the upregulation of IL-18Rα did not differ between CD4^+^25^−^ or CD4^+^25^+^ T cells in culture after activation using a variety of stimulation conditions. However, it is notable that the expression of IL-18Rα was not sustained on the regulatory T cells after day 3 in culture, in contrast to bulk CD4 and CD8 T cell subsets (**Fig. 8**).

## Discussion

This study demonstrates that in immune-deficient mice engrafted with human PBMCs, human IL-18 administration exerts opposite effects upon two human cell populations: CD8^+^ T cell numbers are increased, while CD4^+^CD25^+^FoxP3^+^ Treg cell numbers are markedly decreased. The altered ratio of effector to Treg engraftment was functionally important as judged by the accelerated onset and severity of xenogeneic GVHD in the recipient mice. These effects were unexpected because we are not aware of studies with mouse IL-18 that have revealed differential effects of IL-18 on regulatory and effector T cells [39], [40].

Our studies revealed several potential mechanisms for the effects of IL-18. IL-18 can cause the expansion of mouse NK cells [13], but has not been reported to directly mediate the expansion of T cells. Consistent with this, we were unable to demonstrate a direct effect of IL-18 on the expansion of human effector or regulatory T cells *in vitro*. However, we found that the expression of the IL-18Rα subunit was more prolonged on effector T cells than Tregs, providing a potential mechanism to explain the reciprocal effects that we observed *in vivo* on the effects of IL-18 on effector T cells and Tregs. There may be important species specific differences in the biology of IL-18 on human and mouse T cells [41]. In the mouse, IL-18 has a protective effect on CD4(+)-mediated acute GVHD [42], and blockade of IL-18 accelerates acute GVHD-related mortality in mice [43]. Recent studies in the mouse indicate that IL18 has a role in promoting an IFN-γ dependent positive regulation of memory CD8 T cell proliferation [44]. Recent studies with human cells indicate that the interaction of IL-18 treated NK cells and DCs induces maturation of DCs that subsequently promote Th1 and IFN-γ secretion [45]. Together, the above studies are consistent with our findings, and suggest a mechanism that IL-18 promotes engraftment of CD8 cells in the mice at the expense of regulatory T cells, leading to increased memory CD8 cells and increased xenogeneic GVHD.

A complete mechanistic understanding of the effects mediated by IL-18 is not yet available from the present findings. It is still not clear which T cell subsets are preferentially expanded during IL-18 therapy in the mice, although we found some selective effects on subsets of CD8 T cells in Figure 4. We do not yet know if CD8 T cells need to be activated in order to be regulated by IL-18. Similarly, we do not yet know if CD4 T cells need to be present for the IL-18 effects on CD8 cells. Our data in Figure 6 suggest that the effects on CD8 T cells and Tregs are independent.

IL-18 has been proposed to have a role in a number of inflammatory and autoimmune disorders in mice and man [46]. In mice, IL-18 may have a role in lupus nephritis [47]. In humans, IL-18 over-expression has been reported in rheumatoid arthritis, sarcoidosis, adult-onset Still's disease, vasculitis, lupus, urticaria and histiocytosis [23], [48]–[54]. Tregs have been reported to be decreased or to have decreased functional activity in a number of autoimmune disorders [55], and it is possible that an imbalance of Tregs and effector T cells due to differential IL-18 signaling contributes to the loss of tolerance.

Depletion of Tregs in tumor bearing mice enhances the response to immunotherapy [56]. A high ratio of effector CD8 T cells to Tregs in the tumor microenvironment has been shown to be a favorable prognostic feature in patients with ovarian cancer [57], [58]. An increase in the ratio of CD8^+^ T effector to regulatory cells in syngeneic mouse tumor models and in humans with cancer correlates with responses to immunotherapies [59], [60]. Thus, the ability of IL-18 to increase the ratio of effector CD8^+^ T cells to Tregs may have important implications for therapeutic vaccines and adoptive transfer strategies [61].

The ability of IL-18 to increase the ratio of effector CD8^+^ T cells to Tregs may have important implications for therapeutic vaccines and cancer therapy. Depletion of Tregs in tumor bearing mice enhances the response to immunotherapy [56]. A high ratio of effector CD8 T cells to Tregs in the tumor microenvironment has been shown to be a favorable prognostic feature in patients with ovarian cancer [57], [58]. Furthermore, an increase in the ratio of CD8^+^ T effector to regulatory cells in syngeneic mouse tumor models and in humans with cancer correlates with responses to immunotherapies [59], [60].

Our findings may have important implications for cancer therapy due to the immunosuppressive tumor microenvironment. Cancer patients have increased tumor infiltrating Tregs [62], and in many cases, CD8^+^ CTLs from the tumor environment are dysfunctional [63], [64]. Recently, it has become more widely appreciated that successful immunotherapy requires neutralization of the immunosuppressive features of the tumor microenvironment, in particular Tregs [65]. Thus, by inhibiting Tregs and promoting conventional T cells, human IL-18 may have potential to restore immunocompetence in cancer patients, and thereby augment the effects of cytotoxic and biologic therapies, in order to provoke and maintain an effective anti-tumor immune response. Finally, IL-18 may have potential to augment the anti-tumor effects of donor leukocyte infusions by promoting effector T cells at the expense of regulatory T cell expansion.

## Supporting Information

Figure S1 Photomicrographs of lung and liver tissue sections taken from animals after xenogeneic GVHD induction. 10 million PBMC were transferred into NOG recipient mice followed by daily injections of IL-18 (15 µg) or PBS. Animals were euthanized on day 20 when recipients of PBMC and IL-18 were moribund. Sections were stained with hematoxylin and eosin. Left: Lung sections taken from PBMC or PBMC+IL-18 recipients, demonstrating severe inflammation and lymphocytic infiltration in IL-18 recipients. Right: Liver sections demonstrating marked periportal lymphocytic infiltrates in IL-18 recipients.
(5.49 MB TIF)

Figure S2 Photomicrographs of gut tissue sections taken from animals after xenogeneic GVHD induction. Sections were obtained from the animals described above in Suppl. Fig. S1. Left: low magnification. Middle: higher magnification. Right: highest magnification.
(5.14 MB TIF)
