# Supplementary figures and images for: Distinct Effects of IL-18 on the Engraftment and Function of Human Effector CD8+ T Cells and Regulatory T Cells
Source: PLoS One. 2008 Sep 26;3(9):e3289. doi: 10.1371/journal.pone.0003289 (PMC2538560; doi:10.1371/journal.pone.0003289)

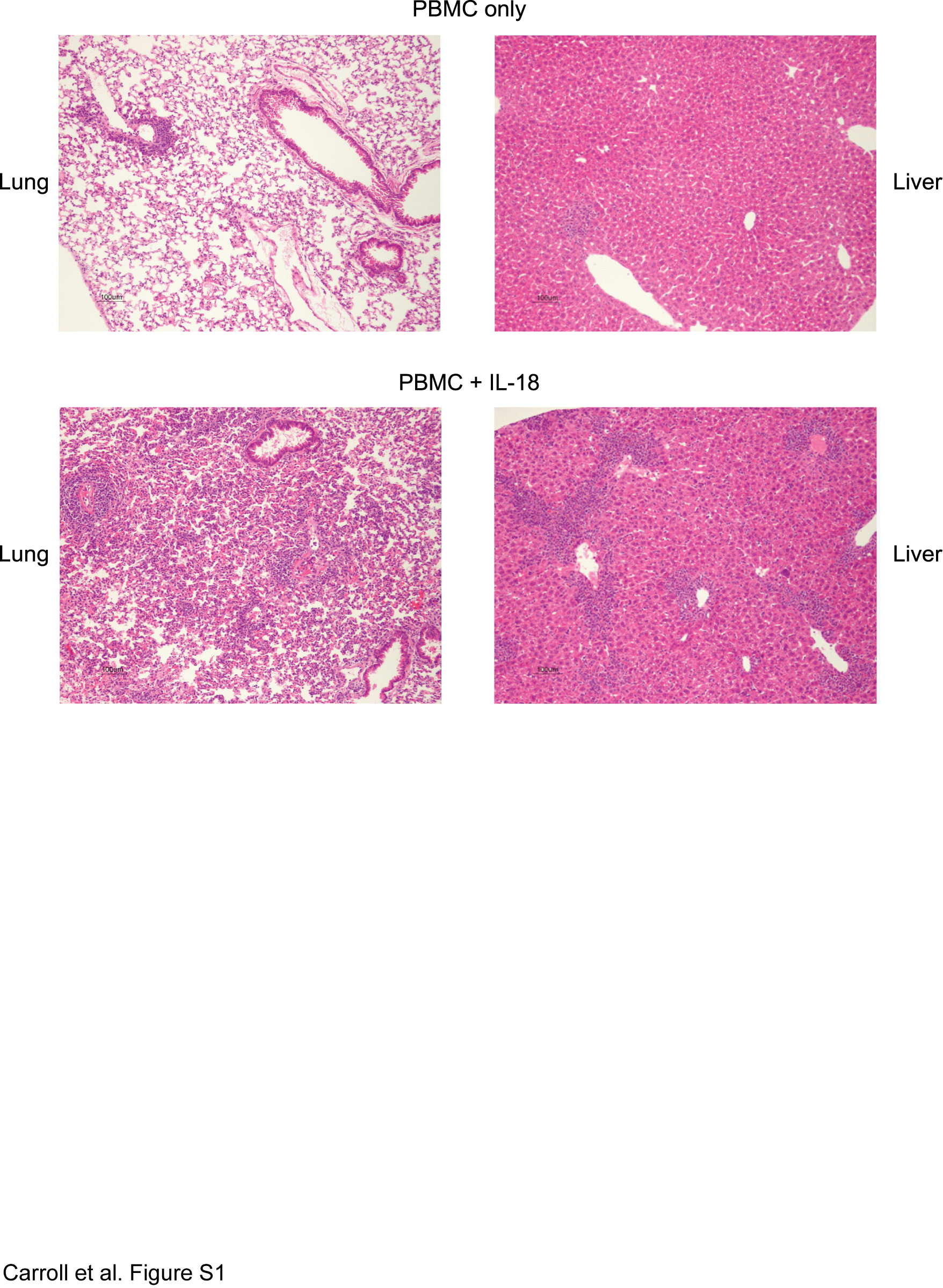

Supplement: Figure S1 — Photomicrographs of lung and liver tissue sections taken from animals after xenogeneic GVHD induction. 10 million PBMC were transferred into NOG recipient mice followed by daily injections of IL-18 (15 µg) or PBS. Animals were euthanized on day 20 when recipients of PBMC and IL-18 were moribund. Sections were stained with hematoxylin and eosin. Left: Lung sections taken from PBMC or PBMC+IL-18 recipients, demonstrating severe inflammation and lymphocytic infiltration in IL-18 recipients. Right: Liver sections demonstrating marked periportal lymphocytic infiltrates in IL-18 recipients. (5.49 MB TIF) [file pone.0003289.s001.tif]

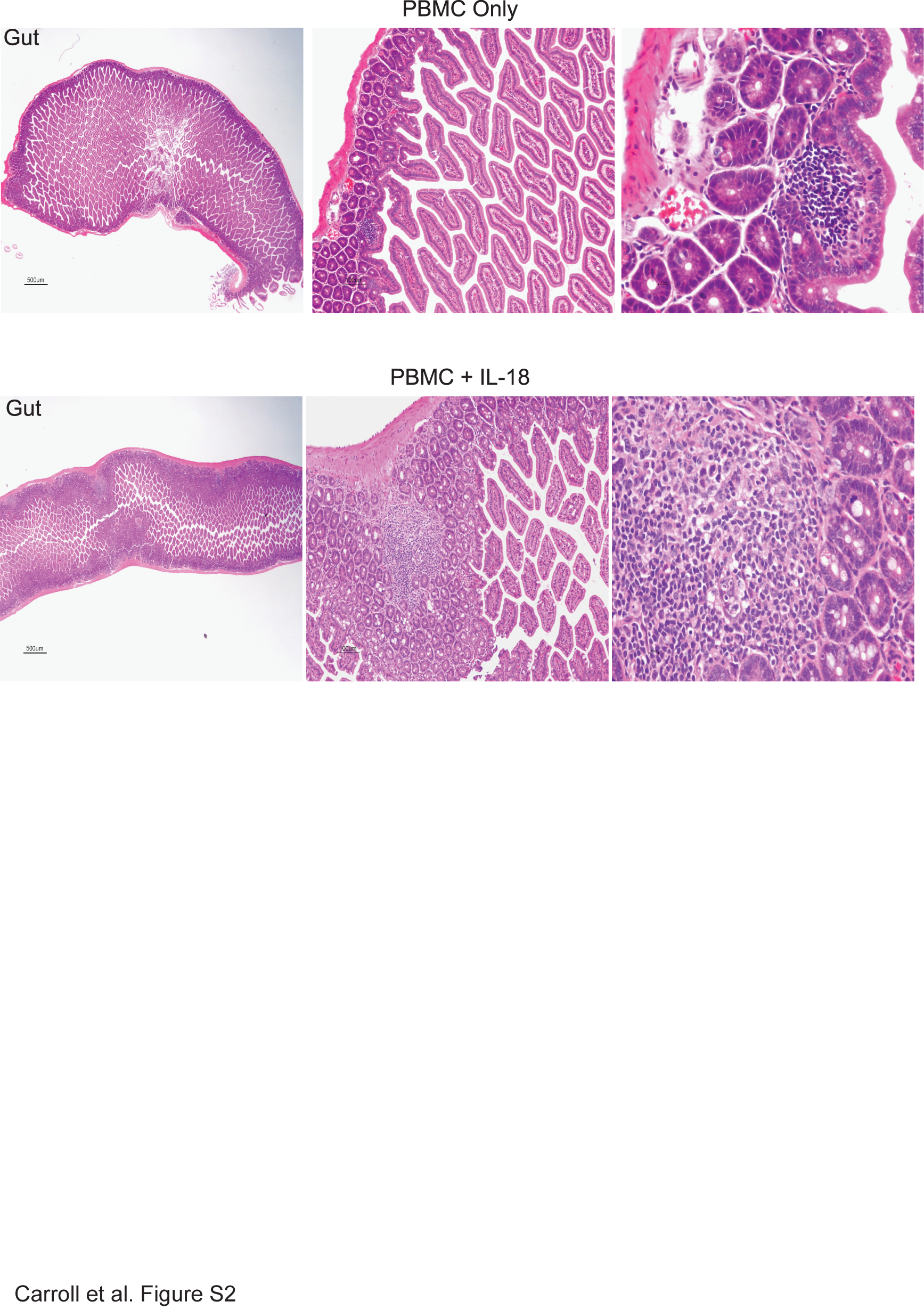

Supplement: Figure S2 — Photomicrographs of gut tissue sections taken from animals after xenogeneic GVHD induction. Sections were obtained from the animals described above in Suppl. Fig. S1. Left: low magnification. Middle: higher magnification. Right: highest magnification. (5.14 MB TIF) [file pone.0003289.s002.tif]
